# Anticholinergic drug exposure is associated with prevalence, worsening and incidence of dysphagia among hospitalized older adults

**DOI:** 10.1016/j.jnha.2025.100507

**Published:** 2025-02-13

**Authors:** Lucia Muglia, Alessia Beccacece, Luca Soraci, Ramona Caloiero, Franco Arturi, Paolo Fabbietti, Mirko Di Rosa, Jacopo Sabbatinelli, Giada Ida Greco, Elvira Filicetti, Mara Volpentesta, Alberto Montesanto, Ersilia Paparazzo, Antonio Cherubini, Massimiliano Fedecostante, Chiara Chinigò, Maria Capalbo, Andrea Corsonello, Fabrizia Lattanzio

**Affiliations:** a Centre for Biostatistics and Applied Geriatric Clinical Epidemiology, Italian National Research Center on Aging (IRCCS INRCA), Cosenza, Italy; b Centre for Biostatistics and Applied Geriatric Clinical Epidemiology, Italian National Research Center on Aging (IRCCS INRCA), Ancona, Italy; c Unit of Geriatric Medicine, Italian National Research Center on Aging (IRCCS INRCA), Cosenza, Italy; d Department of Medical and Surgical Sciences, University "Magna Graecia" Medical School, Internal Medicine Unit, Outpatient Unit for the Treatment of Obesity, University Hospital "R. Dulbecco", 88100 Catanzaro, Italy; e Department of Clinical and Molecular Sciences, Università Politecnica Delle Marche, Ancona, Italy; f Clinic of Laboratory and Precision Medicine, IRCCS INRCA, Ancona, Italy; g Department of Biology, Ecology and Earth Sciences, University of Calabria, Arcavacata di Rende, Italy; h Geriatria, Accettazione Geriatrica e Centro di Ricerca Per L’invecchiamento, IRCCS INRCA, Ancona, Italy; i General Direction, IRCCS INRCA, Ancona, Italy; j Department of Pharmacy, Health and Nutritional Sciences, School of Medicine and Digital Technologies, University of Calabria, Arcavacata di Rende, Italy; k Scientific Direction, IRCCS INRCA, Ancona, Italy

**Keywords:** Dysphagia, Anticholinergic, Older, CALS, ACB.

## Abstract

**Objectives:**

In this study, we aimed at evaluating the association between anticholinergic drug exposure and prevalence, worsening, and incidence of dysphagia among hospitalized older individuals.

**Design and setting:**

We used data from the REPORT-Age project, a multicenter cohort study including patients aged 65 years or more, admitted to acute care hospitals of the Italian National Institute for Health and Sciences on Aging (INRCA-IRCCS) between 2011 and 2019.

**Participants:**

4,005 older patients aged 84.7 (SD = 6.6) years underwent comprehensive geriatric examination according to the interRAI minimum dataset for acute care (MDS-AC), at both hospital admission and discharge.

**Measurements:**

Both prevalence and severity of dysphagia were assessed through items of subjective evaluation included in the section K3 of MDS-AC questionnaire; the anticholinergic drug exposure was measured by means of CRIDECO Anticholinergic Load Scale (CALS) and Anticholinergic Cognitive Burden (ACB) scales. Next, we used logistic regression models to evaluate the association between anticholinergic burden and prevalence of dysphagia at hospital admission; Kaplan Meier cumulative probability curves and cox proportional hazard models were used to analyze the association between anticholinergic burden at hospital admission and worsening or incidence of dysphagia during hospital stay.

**Results:**

Out of 4,005 patients included, 1,070 (30%) presented dysphagia at hospital admission. High anticholinergic burden was associated with increased prevalence of dysphagia at hospital admission (*p* < 0.001). Moreover, patients with high anticholinergic burden (ACB and CALS ≥ 2) were at increased risk of dysphagia worsening during hospital stay (HR, 95%CI: 1.14, 1.06−1.22 and 1.12, 1.03−1.23 for ACB and CALS respectively). Among the 2,935 patients with normal swallowing function at hospital admission, high anticholinergic burden was associated with the incidence of dysphagia at hospital discharge (HR, 95% CI: 1.89, 1.21−2.96 and 1.86, 1.14−3.06 for ACB and CALS respectively). Development of dysphagia during hospital stay was associated with a reduced prescription of anticholinergic medications (ORs, 95% CI 1.13 (1.07–1.20) and 1.08 (1.01–1.15) for ACB and CALS, respectively).

**Conclusions:**

Increased anticholinergic burden was associated with the prevalence, worsening, and incidence of dysphagia among older hospitalized patients.

## Highlights

• This study aims to assess the impact of medium-to-high anticholinergic burden on the risk of dysphagia among an unselected population of hospitalized older individuals
• Both CALS and ACB anticholinergic burden scales were significantly associated with the prevalence, worsening, and incidence of dysphagia among hospitalized older adults.
• The findings support the need for regular medication review to reduce anticholinergic medication use in older adults to mitigate the risk of dysphagia and related complications.

## 1 Introduction

Dysphagia, or difficulty swallowing, is defined as the inability to safely and effectively move the bolus from the mouth to the stomach, either due to problems in the oropharyngeal or esophageal phases of swallowing [1,2]. Oropharyngeal dysphagia is particularly burdening geriatric population and has been recently recognized as a geriatric syndrome [[3], [4], [5]]. Independently of the underlying etiology, diagnosis of dysphagia poses serious health issues when not appropriately managed [2]. Indeed, oral health and good swallowing function are essential for appropriate hydration and oral intake, which are necessary for overall health and survival. Therefore, dysphagia has been associated with increased risk of dehydration, malnutrition, sarcopenia, infections, and aspiration pneumonia [[5], [6], [7], [8], [9]]. The latter has been considered one of the most common causes of death in patients with impaired swallowing capacity. Furthermore, dysphagia has shown to negatively affect physical and psychological well-being and quality of life of older individuals, as well ability in performing activities of daily living (ADLs) [10,11]. For all these reasons, early diagnosis of dysphagia and identification of preventable risk factors for its onset are of utmost importance in the geriatric setting [12].

Numerous factors may contribute to increasing vulnerability of older individuals to develop dysphagia [4,5]. Advanced age may *per se* increase the risk of dysphagia onset even in the absence of underlying chronic conditions. Indeed, older individuals tend to experience a progressive impairment in taste sensitivity and alteration of oropharyngeal sensorimotor function, that predispose them to modifications of swallowing function; additionally, age-related changes in the swallowing process (presbyphagia) and decreased muscle mass (sarcopenic dysphagia) increase the likelihood of dysphagia and its complications in older individuals [13,14]. Furthermore, the concept of oral frailty, encompassing reduced tongue strength, diminished chewing ability, poor dentition, and a decline in salivary flow, has emerged as a critical contributor to the risk of physical frailty and dysphagia in older individuals [15]. Oral frailty does not only affect swallowing function but also exacerbates nutritional deficiencies, aspiration risk, and overall health decline in this population.

Beyond normal aging, several chronic diseases are associated with increased risk of dysphagia in older adults; these include neurological disorders like stroke, Parkinson’s disease, and dementia, as well as chronic obstructive pulmonary disease, heart failure, and gastroesophageal reflux disease [[16], [17], [18]]. Some acute conditions, e.g. delirium [19] and medications may negatively affect the swallowing function, thus increasing the risk for dysphagia. Among drugs, accumulation of anticholinergic medications has emerged as a risk factor for poor survival [20,21], delirium [22], cognitive impairment [23,24], and physical disability [23], among older patients; furthermore, some conditions such as functional dependency [25], depression [26], and low handgrip strength [27] may increase the harms of anticholinergic medications.

Recent studies have also shown that side effects of these medications may potentially alter the swallowing process and lead to increased risk of dysphagia in older people [22,28]. To this regard, the main peripheral anticholinergic side effect, represented by a dose-dependent inhibition of saliva production, may affect the first phases of the swallowing process, thus leading to increased risk of dysphagia [29]; additionally, prolonged use of these drugs may cause malnutrition [30], which may also involve atrophy of the muscles used to swallow, thus potentially exacerbating dysphagia [31]. Although peripheral inhibition of the cholinergic system due to anticholinergic medications may be considered the main contributor for the onset of drug-related dysphagia [29,32], the central effects of anticholinergic medications may lead to cognitive dysfunction and sedation, which can both affect the ability to focus on the swallowing process and further increase the risk for dysphagia or challenge its management [28]. For this reason, considering both peripheral and central anticholinergic effects is essential to capture the overall risk for dysphagia in older patients.

To account for the cumulative risk of taking multiple anticholinergic medications, several scores have been validated to measure the cumulative anticholinergic burden [33]. However, evidence of the association between anticholinergic drug exposure and dysphagia mainly stems from cross-sectional studies in hospitalized older patients [34] and some longitudinal cohort studies in patients with specific disease conditions [22,28]. To fill this gap, we used data from a large observational cohort study including older patients admitted to acute care hospitals, aiming at: (1) evaluating the relationship between measured anticholinergic burden and the prevalence of dysphagia at hospital admission; (2) evaluating the association between anticholinergic burden at hospital admission and the worsening and incidence of dysphagia during the hospital stay; (3) investigating whether diagnosis of dysphagia affected deprescribing practices of anticholinergic medications at hospital discharge.

## 2 Methods

### 2.1 Study population

The present investigation is based on a secondary analysis of the ReportAGE study, a large observational study on outcomes of older patients admitted to acute care hospitals of Italian National Institute of Health and Sciences on Aging (INRCA-IRCCS) [35]. Data collected from acute care wards (geriatric medicine, cardiology, urology, general surgery, and neurology) of three different research hospitals were analyzed. Briefly, all patients consecutively admitted to these wards from September 2011 to December 2019 and able to give informed consent were enrolled in the present study. Criteria for inclusion were age ≥65 years, length of stay more than 24 h, and signed informed consent. Participating physicians and nurses were specifically trained before starting recruitment, as previously described [35]. Information was collected on demographic, socioeconomic, and clinical characteristics, with a detailed evaluation of comprehensive geriatric assessment and pharmacological history; more in detail, patients underwent a comprehensive geriatric assessment (CGA) by Inter-RAI Minimum Data Set acute care (MDS-AC) [36], conducted both at the time of hospitalization and at the time of discharge. Information on medications taken during hospital admission and discharge (including drug name, formulation, and daily dose) was evaluated and coded according to the Anatomical Therapeutic and Chemical classification (ATC).

Overall, 5,935 patients were successfully enrolled during the study period. To the aim of this analysis, we used a sub-sample of 4,005 individuals, obtained after excluding those who died during the hospital stay (n = 573), those with missing data on anticholinergic medications (n = 1165) and those with missing data on swallowing function (n = 192). Patients who died were characterized by a similar anticholinergic burden according to ACB scale (median, IQR score of 1, 0−2 in those who died and in those who survived); in contrast, median CALS score was slightly higher among deceased (2, IQR:1−3) vs. survived (1, 1–2) patients; however, the difference across the 2 groups was not significant even for CALS scores (p value = 0.35).

Analyses on dysphagia’s incidence were performed on a subsample of individuals with normal swallowing function at hospital admission (n = 2,935). The Ethics Committee of the Italian National Center on Aging approved the study protocol (Trial Registration no. NCT01397682). The study was performed in strict accordance with the Helsinki Declaration (Trial Registration no. NCT01397682).

### 2.2 Exposure variables

The exposure variables were built based on the information collected at hospital admission and discharge. In order to evaluate the impact of anticholinergic burden on swallowing function, exposure to anticholinergic medications at hospital admission and discharge has been defined by means of Anticholinergic Cognitive Burden (ACB) scale [37] and CRIDECO Anticholinergic Load Scale (CALS) [38].

The ACB scale was chosen because it was extensively validated in geriatric patients to assess the extent of anticholinergic adverse effects on cognitive function [39]. Drugs with possible anticholinergic effects are defined as those with serum anticholinergic activity or in vitro affinity for muscarinic receptors but with unknown clinically relevant cognitive effects (ACB score 1). Drugs scoring 2 or 3 at ACB scale are those with well-established, clinically relevant cognitive effects, and considered anticholinergic. The ACB score was chosen for this study because it was externally validated [40,41] and it is considered more accurate in the assessment of central anticholinergic burden [42] compared to other tools mainly focused on peripheral anticholinergic effects [43] or aimed at capturing both central and peripheral effects [37].

The CALS was chosen because it was recently developed to update previous scales and contains the largest number of anticholinergic drugs; it has been developed by Ramos et al. after performing a systematic review of the literature and comparing anticholinergic medications included in other 7 scales [38]; in brief, CALS list includes 217 anticholinergic medications classified according to their potency in 3 classes: (a) drugs with low anticholinergic potency (score = 1; 125 medications); (b) drugs with medium anticholinergic potency (score = 2; 28 medications); (c) drugs with high anticholinergic potency (score = 3; 62 medications). The cumulative CALS score was calculated by summing all the burden scores for each drug taken by the patient.

The main exposure variable was calculated at hospital admission and discharge as follows: low anticholinergic burden (ACB or CALS score = 0; no anticholinergic medications); medium (ACB or CALS score = 1); high anticholinergic burden (ACB or CALS scores ≥2). For longitudinal analyses, we the variation of anticholinergic burden between hospital admission and discharge as measured through ACB and CALS was also calculated.

The list of drugs included in the ACB and CALS scales and their distribution at hospital admission in the study population are reported in Supplementary Tables S1 and S2.

### 2.3 Outcome

Assessment of swallowing function was made by using items of MDS-AC 3.0 [36], section K3, related to swallowing function, which was evaluated by physicians and nurses at hospital admission and discharge. These items were coded as follows: 1 = normal swallowing function; 2 = dietary modifications needed to swallow solid meals; 3 = dietary modifications needed to swallow liquid meals; 4 = dietary modifications needed to swallow solid and liquid meals; 5 = combined oral and enteral nutrition with feeding tube; 6 = enteral nutrition with feeding tube; 7 = enteral nutrition with gastro/digiunostomy; 8 = total parenteral nutrition. Dysphagia was defined as having at least need of dietary modification to swallow foods.

Worsening of the swallowing function during hospital stay was also calculated; first we computed the difference between K3 score at discharge and admission; the worsening of swallowing function was defined as an increase of at least 1 point in K3 score from admission to discharge.

Among patients with normal swallowing function at hospital admission, we calculated the incidence of dysphagia at hospital discharge.

### 2.4 Covariates

Age, sex, clinical diagnoses, cognitive function, physical impairment, number of medications prescribed were considered as covariates in the analysis.

Discharge diagnoses were coded by the physician by using International Classification of Diseases, Ninth Revision, Clinical Modification (ICD-9 CM) codes. For each individual diagnosis, a dichotomic analytic variable was calculated. For this study, only diagnoses with a prevalence of at least 5% and capable to impact the swallowing function were included: hypertension, congestive heart failure (HF), coronary artery disease (CAD), atrial fibrillation (AF), cancer, chronic obstructive pulmonary disease (COPD), chronic kidney disease (CKD), diabetes, dementia, and Parkinson’s disease. Finally, the number of prescribed medications was calculated at hospital admission and discharge; medications were coded by using the Anatomical Therapeutic Chemical (ATC) Classification System.

Physical disability was assessed by evaluating the number of Basic Activities of Daily Living (BADLs) impaired at discharge, where impairment was defined as having need of intensive assistance or total dependency in performing each activity. A total of 7 BADL activities were evaluated: bathing, personal hygiene, toilet use, locomotion on unit, transfer, bed mobility and eating.

Cognitive function was evaluated calculating the Cognitive Performance Scale (CFS) based on MDS-AC items [44,45]; possible CPS scores range from 0 (no impairment) to 6 (severe impairment).

### 2.5 Statistical analysis

Firstly, the demographic, clinical, and pharmacological characteristics of the study population were described according to the presence or not of dysphagia at hospital admission. Continuous variables were summarized with mean (standard deviation, SD) when normally distributed and median (interquartile range, IQR) when not. The Kolmogorov–Smirnov test was used to check the normality of study variables. The categorical data were summarized with count and percentages (%). Independent sample t-test, ANOVA, and χ² test were used when appropriate to compare between patients with and without dysphagia. The correlation between ACB and CALS was investigated by using Spearman’s Rho. Cross-sectional association between anticholinergic burden and the likelihood of prevalent dysphagia at hospital admission was evaluated through multivariable logistic regression models, with estimation of odds ratios (ORs) and 95% confidence interval (95%CI). The exposure variable was represented by cumulative anticholinergic burden, measured via CALS or ACB, and it was presented first as a continuous variable and then as a categorical variable according to clinically relevant cut-offs (Anticholinergic score = 0 for low burden; score = 1 for medium burden; score = 2 for high burden). Next, we built two different logistic regression models:

• model A, adjusted for age and sex;
• model B: adjusted for age, sex, number of disabled BADLs, CPS score, clinical diagnoses (hypertension, CHF, CAD, diabetes, atrial fibrillation, cancer, COPD, CKD, stroke, Parkinson’s disease) and number of drugs prescribed at hospital admission.

The longitudinal association between anticholinergic burden and the worsening and incidence of dysphagia was also investigated. The outcomes were reported as cumulative incidences (number and proportion of patients experiencing the event), which were calculated in the whole population and in patients with different anticholinergic burdens. The proportional hazard assumption was tested by using Scheonfeld residuals. Age- and sex-adjusted cumulative probability Kaplan Meier curves with log-rank test were then used to compare the time-to-dysphagia of patients grouped by strata of anticholinergic burden. Therefore, we built bivariate and multivariate cox regression models (models A and B) with hazard ratios (HRs) and 95% CIs to investigate whether anticholinergic burden was associated with incidence of dysphagia during the hospital stay. Sensitivity analyses were conducted in patients belonging to different age groups to evaluate whether study results were different in individuals aged ≥ 85 years and < 85 years.

The exposure variables were represented by either continuous or categorical ACB and CALS scores. Furthermore, to evaluate whether incidence of dysphagia during the hospital stay impacted clinician decision making and deprescribing of anticholinergic medications, we generated a variable defined as the change in anticholinergic burden score between hospital admission (ACB_adm_ and CALS_adm_) and discharge (CALS_dis_ and ACB_dis_). Logistic regression models were then fitted to investigate the associations between the incidence of dysphagia and decreased anticholinergic burden at hospital discharge. All statistical analyses were conducted by using R 4.0 (R Foundation for Statistical Computing, Vienna, Austria, www.r-project.org).

## 3 Results

### 3.1 Descriptive statistics of the study population

Demographic and clinical characteristics of the entire study population, as well as those of patients with and without dysphagia, are reported in Table 1. The study population included 4,005 patients aged 84.7 (SD: 6.6) years, with 41.4% of men, with a median of 3 (0−6) disabled ADL and 3 (1–4) CPS score. The number of prescribed medications at hospital admission was 5 (4−7), with an anticholinergic burden represented by a median ACB of 1 (0−2) and a median CALS of 1 (1−3). The list of anticholinergic medications included in the ACB and CALS classes at hospital admission is reported in Supplementary Tables S1 and S2. The most commonly prescribed medications were represented by furosemide, warfarin, metformin and digoxin among mild anticholinergics, and quetiapine, promazine, and paroxetine among strong anticholinergics; of note, metformin was defined as anticholinergic only in the CALS classification; similarly, some strong anticholinergics had discordant scores in the 2 classifications: indeed, paroxetine, quetiapine, and promazine were scored 2 according to CALS and 3 according to ACB scores (Supplementary Tables S1 and S2).

**Table 1:** Clinical and socio-demographic characteristics of the study population and of patients with and without dysphagia at hospital admission.

	All (n = 4,005)	No dysphagia (n = 2,935)	Dysphagia (n = 1,070)	*p*
Age, mean (SD)	84.7 (6.6)	83.7 (6.5)	87.5 (6.0)	<0.001
Male sex, n (%)	1,660 (41.4)	1,289 (43.9)	471 (34.7)	<0.001
Hypertension, n (%)	1,888 (47.1)	1,469 (50.0)	419 (39.1)	<0.001
CHF, n (%)	521 (13.0)	393 (13.4)	128 (12.0)	0.256
CAD, n (%)	546 (13.6)	435 (14.8)	111 (10.4)	<0.001
Diabetes, n (%)	779 (19.4)	610 (20.8)	169 (15.8)	<0.001
Atrial fibrillation, n (%)	734 (18.3)	539 (18.3)	195 (18.2)	0.956
Cancer, n (%)	489 (12.2)	387 (13.2)	102 (9.5)	0.002
COPD, n (%)	731 (18.2)	574 (19.5)	157 (14.7)	<0.001
CKD, n (%)	1,018 (25.4)	741 (25.2)	277 (25.9)	0.710
Stroke, n (%)	296 (7.4)	210 (7.1)	86 (8.0)	0.381
Dementia, n (%)	1,005 (25.1)	523 (17.8)	482 (45.0)	<0.001
Number of drugs at admission, median (IQR)	5 (4−7)	5 (3.5−7)	6 (4−8)	<0.001
CPS (mean, SD)	2.8 (2.1)	2.1 (1.8)	4.7 (1.7)	<0.001
Number of disabled BADL, median (IQR)	3 (0−6)	1 (0−5)	6 (5−7)	<0.001
ACB, median (IQR)	1 (0−2)	1 (0−2)	1 (0−2)	<0.001
ACB, n (%)				<0.001
0	1,548 (38.6)	1,250 (42.6)	298 (27.8)	
1	1,164 (29.1)	864 (29.4)	300 (28.0)	
≥ 2	1,293 (32.3)	821 (28.0)	472 (44.1)	
CALS, median (IQR)	1 (1−3)	1 (0.2)	2 (1−3)	<0.001
CALS, n (%)				<0.001
0	925 (23.1)	754 (25.7)	171 (16.0)	
1	1,124 (28.1)	877 (29.9)	247 (23.1)	
≥ 2	1,956 (48.8)	1,304 (44.4)	652 (60.9)	
Concordance, n (%)				<0.001
ACB = CALS	2,211 (55.2)	1,668 (56.8)	543 (50.7)	
ACB > CALS	277 (6.9)	145 (4.9)	132 (12.3)	
CALS > ACB	1,517 (37.9)	1,122 (38.2)	395 (50.7)	

Notes: ACB = anticholinergic cognitive burden; BADL = basic activities of daily living; CAD = coronary artery disease; CALS = CRIDECO anticholinergic load scale; CHF = congestive heart failure; CKD = chronic kidney disease; COPD = chronic obstructive pulmonary disease; CPS = cognitive performance scale; IQR = interquartile range.

Although a very high concordance was found between the two scales (Spearman’s rho: 0.79), patients with high anticholinergic burden (score ≥ 2) were more commonly identified by CALS (48.8% vs. 32.3%, *p* < 0.001). This mainly derived by accumulation of drugs with low-medium anticholinergic properties, given the relatively low prescription rate of drugs categorized as CALS = 3 (Supplementary Table S2).

### 3.2 Risk factors for the prevalence of dysphagia at hospital admission

Out of 4,005 patients, 1,070 presented dysphagia at hospital admission, with a prevalence rate of 30%. Patients with dysphagia were older, more commonly women, and had a poorer BADL and CPS performance; furthermore, they took a higher number of overall and anticholinergic medications, with a greater representation of high anticholinergic classes (≥2) compared to patients without dysphagia. They also had a significantly higher prevalence of dementia, and a lower prevalence of hypertension, CAD, diabetes, cancer, and COPD (Table 1). High anticholinergic burden was more prevalent in patients with dysphagia compared to those with normal swallowing function (*p* < 0.001). Differences in the prevalence of anticholinergic medications in the two groups are reported in Supplementary Table S3. In summary, patients with dysphagia had a higher prescription of furosemide and levodopa/carbidopa among mild anticholinergics, as well as quetiapine and promazine among strong ones. Conversely, the prescription of metformin was lower in patients with dysphagia. Logistic regression models evaluating the association between anticholinergic burden and prevalence of dysphagia at hospital admission are reported in Supplementary Table S4: the high anticholinergic burden, which was identified by having either CALS or ACB score ≥2, was associated with dysphagia in both bivariate and multivariate regression models. The association was confirmed when using continuous ACB and CALS scores.

### 3.3 Longitudinal association between anticholinergic burden and worsening of swallowing function

**Fig. 1:**
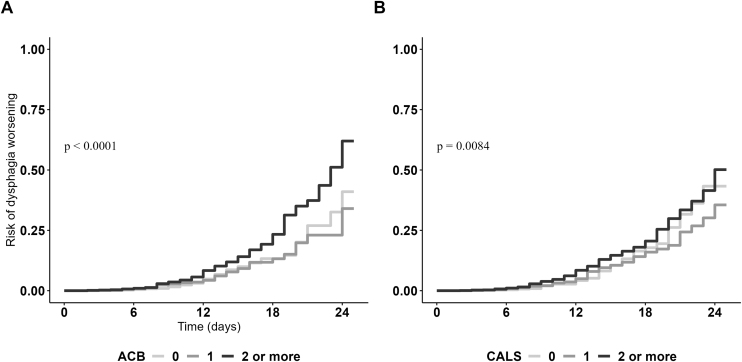
Kaplan Meier cumulative probability curves showing the association between levels of anticholinergic scales and dysphagia worsening.

During a median hospital stay of 9 days, 267 out of 4,005 patients (6.5%) worsened their swallowing functioning. The time-to-event Kaplan Meier cumulative probability curves showing the association between CALS and ACB categories at hospital admission and dysphagia worsening are reported in Fig. 1. Interestingly, both higher ACB and CALS scores were associated with increased probability of dysphagia worsening during hospital stay; however, the curves significantly diverged in the transition between low-medium and high anticholinergic burden for both ACB and CALS scores, despite this difference was more evident for ACB scale compared to CALS; these results were further confirmed by cox regression models shown in Table 2. Indeed, continuous ACB and CALS scores were both associated with increased risk of dysphagia worsening during hospital stay in bivariate and multivariate cox regression models. Furthermore, patients with ACB and CALS scores of 2 or more at hospital admission presented an increased risk of dysphagia worsening.

**Table 2:** Cox Regression analysis showing the association between anticholinergic scales and worsening of dysphagia during hospital stay.

Exposure variable		Model A, HR (95%CI)	Model B, HR (95%CI)
ACB score		1.15 (1.07−1.23)	1.14 (1.06−1.22)
CALS score		1.11 (1.03−1.19)	1.12 (1.03−1.23)
ACB categories			
	0 (ref)	–	–
	1	0.94 (0.68−1.30)	1.11 (0.78−1.56)
	≥ 2	1.39 (1.05−1.86)	1.51 (1.08−2.09)
CALS categories			
	0 (ref)	–	–
	1	1.01 (0.68−1.49)	1.14 (0.75−1.71)
	≥ 2	1.45 (1.03−2.03)	1.55 (1.05−2.28)

Model A: age- and sex-adjusted.

Model B: Model A + CPS + impaired BADL + number of medications at hospital admission + chronic diseases (hypertension, CHF, CAD, diabetes, atrial fibrillation, cancer, COPD, CKD, stroke, Parkinson’s disease).

**Fig. 2:**
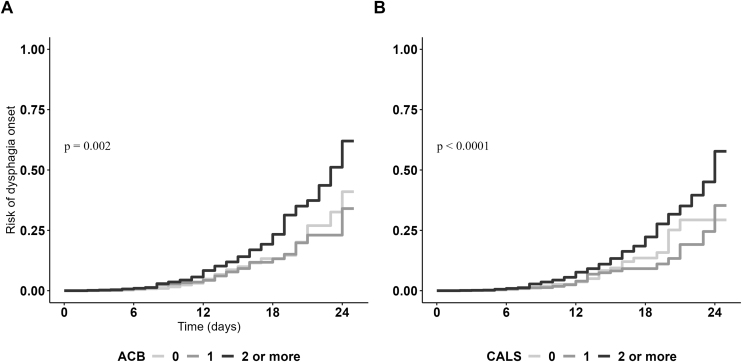
Kaplan Meier cumulative probability curves showing the association between anticholinergic levels and dysphagia incidence.

Among the 2,935 patients with normal swallowing function at hospital admission, 157 (5.4%) developed dysphagia during hospitalization. As shown in Fig. 2, the graded increase in ACB categories was associated with a progressively higher risk of dysphagia onset. In contrast, CALS score presented a less graded association with incidence of dysphagia. Survival analyses showed that high anticholinergic burden measured through both scales (CALS or ACB ≥ 2) was associated with increased risk of new-onset dysphagia (Table 3). Models using continuous rather than categorical scores confirmed the statistically significant association. Interestingly, the increased anticholinergic burden between hospital admission and discharge was not associated with the incidence of dysphagia (Supplementary Table S5). Conversely, logistic regression showed that the incidence of dysphagia was associated with decreased prescription of anticholinergic medications at hospital discharge (fully adjusted ORs, 95% CI 1.13, 1.07–1.20 for ACB deprescribing and 1.08, 1.01–1.15 for CALS deprescribing).

**Table 3:** Cox regression models showing the association of ACB and CALS scores with the incidence of dysphagia at hospital discharge.

Exposure variable	Model A, HR (95%CI)	Model B, HR (95%CI)
ACB score	1.23 (1.13−1.34)	1.19 (1.08−1.30)
CALS score	1.19 (1.08−1.31)	1.20 (1.06−1.36)
ACB categories		
0 (ref)	–	–
1	1.03 (0.68−1.56)	1.26 (0.80−2.00)
≥ 2	1.77 (1.22−2.56)	1.89 (1.21−2.96)
CALS categories		
0 (ref)	–	–
1	0.81 (0.49−1.35)	1.01 (0.59−1.74)
≥ 2	1.65 (1.08−2.52)	1.86 (1.14−3.06)

Notes: ACB = anticholinergic cognitive burden; CALS = CRIDECO anticholinergic load scale.

Model A: age- and sex-adjusted.

Model B: Model A + CPS + impaired BADL + number of medications at hospital admission + chronic diseases (hypertension, CHF, CAD, diabetes, atrial fibrillation, cancer, COPD, CKD, stroke, Parkinson’s disease).

All analyses were also performed in patients aged ≥85 years and <85 years to evaluate whether individual’s age at hospital admission might modify the observed findingsIndividuals aged ≥ 85 years were characterized by a higher prevalence of dysphagia (35.2% vs. 16.9%, *p value* <0.001) and a slightly higher number of medications (median 6, IQR:3−7) compared to younger patients (5, 3–7, p value 0.026); conversely, anticholinergic burden was similar between the two groups when using ACB (1, 0−2 for both groups) and slightly higher in older patients when using CALS score (2, 1−3 vs. 1, 0−2, *p* < 0.001). Sensitivity analyses showing the association between anticholinergic burden and prevalence, worsening, and incidence of dysphagia in the two age groups are reported in Supplementary Tables S6–S8. As regards the association between anticholinergic burden and prevalence of dysphagia, the stratification did not substantially change study results, even if its strength was higher among those aged < 85 years compared to older patients; on the other hand, association between continuous anticholinergic scores and either worsening or incidence of dysphagia during hospital stay was statistically significant only among patients aged ≥85 years; however, a trend of nonsignificant association between increasing anticholinergic exposure and study outcomes was maintained among individuals aged <85 years.

## 4 Discussion

In this study including a large cohort of hospitalized older patients, we provide evidence of the association between high anticholinergic burden and prevalence, worsening, and incidence of dysphagia. Both CALS and ACB scores predicted study outcomes, despite associations were statistically significant especially among individuals ≥85 years. Previous studies showed that preliminary evidence about the association between anticholinergic burden and dysphagia was limited [22,34,46]. Indeed, the only longitudinal studies were conducted in convalescent older patients with stroke [22] and in a small cohort of Japanese patients with tube feeding placement [46].

Anticholinergic medications can affect the swallowing function through several mechanisms. These drugs can cause the inhibition of peripheral muscarinic receptors with subsequent decrease in saliva production and development of dry mouth (xerostomia) and swallowing dysfunction [29,47]. Decreased saliva production is often accompanied by anticholinergic-induced stimulation of thirst sensation which becomes detrimental in patients with impaired swallowing function, potentially leading to aspiration pneumonia [48] and further worsening of dysphagia because of pneumonia-associated sarcopenia [49]. Moreover, some anticholinergics can cause undernutrition which can significantly decrease oropharyngeal muscle mass, strength and worsen presbyphagia and oropharyngeal swallowing dysfunction [50]; such effects can be precipitated by weakening of oropharyngeal muscles due to presbyphagia and sarcopenia, which explain part of the increased susceptibility of older adults to anticholinergic medications [51]. Furthermore, aging itself induces pharmacokinetic and pharmacodynamic changes, as well as increased blood-brain-barrier permeability, decreased cholinergic reserve and brain muscarinic receptor density, which altogether make older individuals particularly vulnerable to anticholinergic side effects [52]. Interestingly, the cognitive decline and sedation induced by anticholinergic drug accumulation may decrease attention and motivation to eat food, with subsequent poor swallowing capacity. However, in our cohort, relationships between anticholinergic burden and occurrence of dysphagia remained consistent even after correcting for cognitive and physical impairment, thereby underlining the solidity of study findings.

Despite most of the detrimental effects of anticholinergic medications were shown to be cumulative and dose-dependent, some drugs may particularly dangerous for people at risk of dysphagia; for instance dopamine antagonists like metoclopramide can lead to dyskinesia and dystonia which may alter oropharyngeal muscle movements [53]; benzodiazepines can lead to minimal sedation but with partial inhibition of swallowing and cough reflex [54]; typical and atypical antipsychotics can cause extrapyramidal disorders that interfere with mouth and muscle movements [55], and decrease the pharingolaryngeal release of substance P, which cause impairment in swallowing reflexes [56]. In this regard, the results of the present study confirmed the dangerous effect of both cumulative anticholinergic burden and prescription of individual detrimental anticholinergic medications; it is not surprising that highly risky medications like promazine, paroxetine, and quetiapine were more commonly prescribed to patients with dysphagia. In our cohort, despite a relatively high concordance between the ACB and CALS scores, the latter identified a higher proportion of patients with high anticholinergic burden; however, the increased risk for dysphagia occurrence was detected starting from anticholinergic burden scores ≥2. These findings underline the importance of reviewing polypharmacy regimens in hospitalized older patients because of their increased susceptibility to developing swallowing disorders and dysphagia. Indeed, although older patients are more vulnerable to anticholinergic side effects, these drugs are commonly prescribed worldwide; their prevalence is reported to be 15–50% in community-dwelling older individuals and to reach 80% of older hospitalized patients [[57], [58], [59]]. Especially in hospitalized older patients, rates of multimorbidity and polypharmacy are particularly high, and the probability of exposure to anticholinergic medications increases significantly [[60], [61], [62]]. With the global population aging, occurrence of dysphagia is expected to rise, highlighting the importance of awareness and proper management of this critical condition in geriatric care. Multidisciplinary assessment of swallowing function during hospital stay should involve multiple professional figures including nurses, physiotherapists, and physicians; nurse routinary use of MDS-AC scales to screen for swallowing disorders and anticholinergic burden scales may allow an easier identification of patients at risk and deserving a thorough objective evaluation of swallowing function. Furthermore, revising anticholinergic burden scales and polypharmacy regimens may help physicians to identify potentially dangerous medications early and initiate deprescribing practices. In the present study, occurrence of dysphagia was associated with a decreased anticholinergic burden from hospital admission to discharge; deprescribing of anticholinergics may be particularly important and should be preferred over modifying oral medications, which can instead affect the safety and efficacy of the administered drug, and has previously shown to be common in older patients with dysphagia [63]; conversely, increased anticholinergic burden from admission to discharge was not associated with increased incidence of dysphagia or dysphagia worsening; the lack of association may be due to the fact that long durations of exposure to anticholinergic effects are needed before any alteration in swallowing ability can be observed; therefore, an increase in the dose or number of anticholinergic drugs between admission and discharge may not be sufficient to induce pharmacodynamic effects capable of altering the swallowing response. Further studies are needed to evaluate the effects of deprescribing interventions on partial or full recovery of swallowing function in older patients with dysphagia.

Our study has several limitations: given the observational study design, confounding by indication and potential non-anticholinergic effects of drugs classified as anticholinergics might have affected study results; additionally, we could not account for illness severity and duration which may influence the association of anticholinergic medications and study outcomes; moreover, the analysis did not account for the duration of anticholinergic medication use or whether medications were prescribed immediately prior to hospital admission. As a result, we were unable to differentiate between long-term use and acute or short-term prescriptions, which might have distinct effects on outcomes such as swallowing function or other clinical parameters; furthermore, assessment of dysphagia was mainly based on subjective evaluation, and speech and language pathologist referral was not systematically asked for, which may have led to underestimated prevalence at hospital admission and discharge; finally, our results apply to a population of older patients discharged from acute care hospitals and cannot be generalized to the general older population. Nevertheless, the strengths of our study are substantial: to the best of our knowledge, we provide first evidence of the association between increased anticholinergic burden and the prevalence, worsening, and incidence of dysphagia in an unselected population of older hospitalized patients; preliminary findings of cross-sectional analyses were confirmed in longitudinal models, which minimize the effects of unmeasured confounders, thus reinforcing the observed associations; furthermore, the detailed assessment of medications and the comprehensive geriatric examination allowed us to explore the independent effect of anticholinergic scores after adjusting for a wide set of potential confounders.

## 5 Conclusions and implications

In conclusion, our findings suggest that an increased anticholinergic burden as measured according to the ACB and CALS scores is linked to the prevalence, worsening, and occurrence of dysphagia in older hospitalized patients. Additionally, there is a significant trend in deprescribing anticholinergic medications in patients who developed or experienced worsening dysphagia during hospital stay. Given the significant impact of dysphagia on the geriatric population, future prospective studies are needed to confirm these findings and to develop strategies for managing the risk of dysphagia associated with the excessive use of anticholinergic medications.

## CRediT authorship contribution statement

Conceptualization: L.S, L.M., A.B., A.Co, F.L.; Data curation and Formal analysis: L.S, P.F.; Investigation: L.S., A.Co.; Methodology: L.S, P.F., M.D.R., A.Co., F.L.; Supervision: L.S., A.Ch, A.Co, F.L.; Roles/Writing - original draft: L.S, L.M., P.F., A.B., A.Co.; and Writing - review & editing: all authors. All authors interpreted the results, commented on and revised successive drafts of the manuscript, and approved the final version

## Ethics approval and consent to participate

The Ethical Committee of the Italian National Research Center on Aging has approved the study protocol which was in accordance with 1964 Helsinki declaration. The trial registration number is NCT01397682. Signed informed consent was obtained from all participants in the study.

## Declaration of Generative AI and AI-assisted technologies in the writing process

I declare that I have not used any generative AI or AI-assisted technologies in the writing process

## Funding

The Report-AGE study (NCT01397682) is partially supported by the Italian Ministry of Health (Grant n. RF-2013–02358848) and Rete Aging (Fondi Ricerca Corrente Reti). The present research was also supported by the “Functional Foods Italy Network” (project code T5-AN-11), funded under the Public Notice of the Italian Ministry of Health on June 7, 2021, within Trajectory 5: “Nutraceuticals, Nutrigenomics, and Functional Foods”, Action 5.1: “Creation of an action program for combating malnutrition and promoting the Mediterranean diet” of the Operational Health Plan.

## Availability of data

Anonymized data and code used in conducting the analyses will be made available upon request directed to the corresponding author.

## Declaration of competing interest

The authors declare that they have no known competing financial interests or personal relationships that could have appeared to influence the work reported in this paper.
