# Supplementary material for: Anticholinergic drug exposure is associated with prevalence, worsening and incidence of dysphagia among hospitalized older adults
Source: J Nutr Health Aging. 2025 Feb 13;29(5):100507. doi: 10.1016/j.jnha.2025.100507 (PMC12180006; doi:10.1016/j.jnha.2025.100507)
Supplement: Supplementary file 1 [file mmc1.doc]

**Supplementary Table 1**. Distribution of drugs with anticholinergic properties according to Anticholinergic Cognitive Burden (ACB) and CRIDECO Anticholinergic Load Scale (CALS) measured at hospital admission

| ACB=1 | % | ACB=2 | % | ACB=3 | % |
| --- | --- | --- | --- | --- | --- |
| **Alimemazine** | **0** | **Amantadine** | 0.1 | **Amitriptyline** | 0.4 |
| **Alprazolam** | **3.5** | *Belladonna alkaloids ↓* | <0.1 | **Amoxapine** | 0.0 |
| **Alverine** | **0** | **Carbamazepine** | 0.6 | **Atropine** | 0.0 |
| **Atenolol** | **1.9** | **Cyclobenzaprine** | 0.0 | **Benzatropine** | 0.0 |
| **Bupropion** | 0.1 | **Cyproheptadine** | 0.0 | **Brompheniramine** | 0.0 |
| **Captopril** | <0.1 | **Loxapine** | 0.0 | **Carbinoxamine** | 0.0 |
| **Chlorthalidone** | <0.1 | *Levomepromazine ↓* | <0.1 | **Chlorphenamine** | <0.1 |
| *Cimetidine ↓* | 0 | **Meperidine** | 0.0 | **Chlorpromazine** | <0.1 |
| **Clorazepate** | <0.1 | **Molindone** | 0.0 | **Clemastine** | 0.0 |
| **Codeine** | <0.1 | **Oxcarbazepine** | 0.3 | **Clomipramine** | <0.1 |
| Colchicine | 0.1 | **Pimozide** | 0.0 | **Clozapine** | 0.1 |
| **Diazepam** | 1.5 |  |  | **Darifenacin** | 0.0 |
| **Digoxin** | 7.2 |  |  | **Desipramine** | 0.0 |
| **Dipyridamole** | 0 |  |  | **Dicyclomine** | 0.0 |
| **Disopyramide phosphate** | 0 |  |  | Dimenhydrinate | 0.0 |
| **Fentanyl** | 0 |  |  | **Doxepin** | 0.0 |
| **Fluvoxamine** | <0.1 |  |  | **Flavoxate** | <0.1 |
| **Furosemide** | 36.1 |  |  | **Hydroxyzine** | <0.1 |
| **Haloperidol** | **1.3** |  |  | **Hyoscyamine** | 0.0 |
| **Hydralazine** | **0** |  |  | **Imipramine** | 0.0 |
| **Hydrocortisone** | **0.1** |  |  | **Meclozine** | 0.0 |
| **Isosorbide mononitrate** | 0.1 |  |  | **Nortriptyline** | 0.0 |
| **Isosorbide dinitrate** | 3.5 |  |  | *Olanzapine ↑* | 1.6 |
| **Loperamide** | <0.1 |  |  | **Orphenadrine** | <0.1 |
| **Metoprolol** | 6.4 |  |  | **Oxybutynin** | 0.1 |
| **Morphine** | <0.1 |  |  | *Paroxetine ↑* | 1.7 |
| **Nifedipine** | 1.3 |  |  | *Perphenazine ↑* | 0.0 |
| **Prednisone** | 1.8 |  |  | **Procyclidine** | 0.0 |
| **Quinidine** | 0.0 |  |  | *Promazine ↑* | 3.8 |
| *Ranitidine ↓* | 0.6 |  |  | **Promethazine** | <0.1 |
| **Risperidone** | 1.7 |  |  | **Propantheline** | 0.0 |
| **Theophylline** | 0.5 |  |  | **Pyrilamine** | 0.0 |
| **Trazodone** | 4.3 |  |  | *Quetiapine ↑* | 6.7 |
| **Triamterene** | 0.0 |  |  | **Scopolamine** | 0.2 |
| **Warfarin** | 11.0 |  |  | **Thioridazine** | 0.0 |
|  |  |  |  | **Tolterodine** | <0.1 |
|  |  |  |  | **Trifluoperazine** | 0.0 |
|  |  |  |  | **Trihexyphenidyl** | 0.0 |
|  |  |  |  | **Trimipramine** | <0.1 |
|  |  |  |  |  |  |

**Supplementary Table 2.** Distribution of drugs with anticholinergic properties according to CRIDECO anticholinergic load scale (CALS) measured at hospital admission (**drugs in bold are those included in both ACB and CALS with the same score, in italic those included in both scales but with different scores ).**

| CALS=1 | % | CALS=1 | % | CALS=2 |  | CALS=3 |  |
| --- | --- | --- | --- | --- | --- | --- | --- |
| Aclidinium | 0.2 | Ketorolac | 0.2 | **Amantadine** | <0.1 | Acepromazine | 0 |
| **Alimemazine** | **0** | Ketotifen | 0 | Baclofen | 0.3 | **Amitriptyline** | 0.4 |
| **Alprazolam** | **3.5** | Levocetirizine | 0 | **Carbamazepine** | 0.6 | **Amoxapine** | 0 |
| **Alverine** | **0** | Levodopa-carbidopa | 5.5 | Cloperastine | 0 | **Atropine** | 0 |
| Amisulpride | 0.2 | Lithium | <0.1 | *Cimetidine↑* | 0 | *Belladonna alkaloids ↑* | <0.1 |
| Ampicillin | <0.1 | **Loperamide** | <0.1 | **Cyclobenzaprine** | 0 | **Benzatropine** | 0 |
| Aripiprazole | 0.2 | Loratadine | 0 | Dosulepin | 0 | Biperiden | 0.3 |
| Asenapine | 0 | Lorazepam | 4.6 | Fluphenazine | 0 | **Brompheniramine** | 0 |
| **Atenolol** | **1.9** | Lumiracoxib | 0 | **Loxapine** | 0 | **Carbinozamine** | 0 |
| Azathioprine | <0.1 | Mebeverine | <0.1 | Maprotiline | 0 | Carisoprodol | 0 |
| Benazepril | 0 | Metformin | 9.0 | **Meperidine** | 0 | **Chlorphenamine** | <0.1 |
| Betaxolol | 0 | Methocarbamol | 0 | Methadone | 0 | **Chlorpromazine** | <0.1 |
| Bisacodyl | <0.1 | Methotrexate | 0 | **Molindone** | 0 | Chlorprothixene | 0 |
| Bromocriptine | <0.1 | Methylprednisolone | 4.3 | Nefopam | 0 | Cimetropium bromide | 0 |
| Bromperidol | <0.1 | Metoclopramide | 0.4 | *Olanzapine ↓* | 1.6 | **Clemastine** | 0.0 |
| **Bupropion** | 0.1 | **Metoprolol** | 6.4 | **Oxcarbazepine** | 0.3 | **Clomipramine** | <0.1 |
| **Captopril** | <0.1 | Midazolam | 0 | *Paroxetine ↓* | 1.7 | **Clozapine** | 0.1 |
| Cefamandol | 0 | Mirtazapine | 1.6 | *Perphenazine ↓* | 0 | Cyproheptadine | 0 |
| Cefoxidin | 0 | **Morphine** | <0.1 | **Pimozide** | 0 | **Darifenacin** | 0.0 |
| Celecoxib | 0.2 | Naratriptan | 0 | Prochlorperazine | 0 | **Desipramine** | 0.0 |
| Cephalothin | 0 | Nefazodone | 0 | *Promazine ↓* | 3.8 | Dexbrompheniramine | 0 |
| Cetirizine | 0.3 | **Nifedipine** | 1.3 | Propoxyphene | 0 | Dexchlorpheniramine | 0 |
| Cinnarizine | <0.1 | Nizatidine | 0 | *Quetiapine↓* | 6.7 | **Dicyclomine** | 0.0 |
| Chlordiazepoxide | 0 | Oxazepam | <0.1 | *Ranitidine ↑* | 0.6 | Difemerine | 0 |
| **Chlorthalidone** | <0.1 | Oxycodone | 0.1 | Tramadol | 0.6 | Diphenhydramine | 0 |
| Citalopram | 1.2 | Paliperidone | 0 | Triprolidine | 0 | **Doxepin** | 0.0 |
| Clindamycin | <0.1 | Pancuronium | 0 | Zotepine | 0 | Doxylamine | 0 |
| Clonazepam | 0.9 | Phenelzine | 0 | Zuclopenthixol | <0.1 | Emepronium | 0 |
| **Clorazepate** | <0.1 | Phenobarbital | 1.6 |  |  | Fesoterodine | 0 |
| **Codeine** | <0.1 | Piperacillin | 0.5 |  |  | **Flavoxate** | <0.1 |
| Cortisone | 0.2 | Pramipexol | 0.7 |  |  | Homatropine | 0 |
| Cycloserine | 0 | Prednisolone | 0 |  |  | **Hydroxyzine** | <0.1 |
| Cyclosporine | <0.1 | **Prednisone** | 1.8 |  |  | **Hyoscyamine** | 0 |
| Desloratadine | 0 | Pridinol | 0 |  |  | **Imipramine** | 0 |
| Desvenlafaxine | 0 | Pseudoephedrine | 0 |  |  | *Levomepromazine↑* | <0.1 |
| Dexamethasone | 0.3 | **Quinidine** | 0 |  |  | **Meclozine** | 0 |
| Dextromethorphan | 0 | **Risperidone** | 1.7 |  |  | Mequitazine | 0 |
| **Diazepam** | 1.5 | Rotigotine | 1.0 |  |  | **Nortriptyline** | 0 |
| Digitoxin | 0 | Selegiline | 0.5 |  |  | Opipramol | 0 |
| **Digoxin** | 7.2 | Sertraline | 2.5 |  |  | **Orphenadrine** | <0.1 |
| Diltiazem | 1.2 | Sumatriptan | 0 |  |  | Otilonium bromide | <0.1 |
| **Dipyridamole** | 0 | Tapentadol | <0.1 |  |  | **Oxybutynin** | 0.1 |
| **Disopyramide phosphate** | 0 | Temazepam | 0 |  |  | Pheniramine | 0 |
| Domperidone | 1.6 | **Theophylline** | 0.5 |  |  | **Procyclidine** | 0 |
| Entacapone | 0 | Tiotixene | 0 |  |  | **Promethazine** | <0.2 |
| Escitalopram | 4.0 | Tiotropium | 4.8 |  |  | **Propantheline** | 0 |
| Estazolam | 0.1 | Trandolapril | <0.1 |  |  | Propiverine | 0 |
| Famotidine | 0 | **Trazodone** | 4.3 |  |  | Protriptyline | 0 |
| **Fentanyl** | 0 | Triamcinolone | <0.1 |  |  | **Pyrilamine** | 0 |
| Fexofenadine | 0 | **Triamterene** | 0 |  |  | **Scopolamine** | 0.2 |
| Flunitrazepam | 0 | Trimebutine | <0.1 |  |  | Solifenacin | 0 |
| Flupentixol | 0 | Triazolam | 1.0 |  |  | **Thioridazine** | 0 |
| Fluoxetine | <0.1 | Umeclidinium | <0.1 |  |  | Tiemonium iodide | 0 |
| Flurazepam | 0.2 | Valproic acid | 1.0 |  |  | Timepidium bromide | 0 |
| **Fluvoxamine** | <0.1 | Vancomycin | <0.1 |  |  | Tizanidine | 0.2 |
| **Furosemide** | 36.1 | Venlafaxine | 2.4 |  |  | **Tolterodine** | <0.1 |
| Gentamicin | 0.1 | **Warfarin** | 11.0 |  |  | **Trifluoperazine** | 0.0 |
| Glycopirronium | 0.2 | Ziprasidone | 0 |  |  | **Trihexyphenidyl** | 0.0 |
| Guaifenesin | 0 | Zolmitriptan | 0 |  |  | **Trimipramine** | <0.1 |
| **Haloperidol** | **1.3** |  |  |  |  | Tropatepine | 0 |
| **Hydralazine** | **0** |  |  |  |  | Trospium | 0 |
| Hydrocodone | 0.1 |  |  |  |  | Valethamate | 0 |
| **Hydrocortisone** | **0.1** |  |  |  |  |  |  |
| Hydromorphone | 0 |  |  |  |  |  |  |
| Iloperidone | 0 |  |  |  |  |  |  |
| Ipratropium | 0.2 |  |  |  |  |  |  |
| **Isosorbide mononitrate** | 0.1 |  |  |  |  |  |  |
| **Isosorbide dinitrate** | 3.5 |  |  |  |  |  |  |

**Supplementary Table 3. Differences in the prescription of most commonly prescribed anticholinergic medications in patients with and without dysphagia at hospital admission**

| **Medications, n (%)** | **Anticholinergic score** | **Not dysphagia** | **Dysphagia** | ***P value*** |
| --- | --- | --- | --- | --- |
| Furosemide | ACB and CALS=1 | 987 (33.6) | 459 (42.9) | <0.001 |
| Warfarin | ACB and CALS=1 | 322 (11.0) | 118 (11.0) | 0.999 |
| Metformin | Exclusively CALS=1 | 295 (10.0) | 66 (6.2) | <0.001 |
| Digoxin | ACB and CALS=1 | 203 (6.9) | 89 (8.3) | 0.150 |
| Metoprolol | ACB and CALS=1 | 185 (6.3) | 71 (6.6) | 0.758 |
| Levodopa/carbidopa | Exclusively CALS=1 | 119 (4.0) | 100 (9.3) | <0.001 |
| Quetiapine | ACB=3, CALS=2 | 132 (4.5) | 136 (12.7) | <0.001 |
| Promazine | ACB=3, CALS=2 | 63 (2.1) | 91 (8.5) | <0.001 |
| Paroxetine | ACB=3, CALS=2 | 56 (1.9) | 14 (1.3) | 0.252 |

**Supplementary Table 4: logistic regression analysis showing associations between anticholinergic scales and prevalence of dysphagia at hospital admission**

|  | **Model A*** | **Model B**** |
| --- | --- | --- |
| **ACB score** | 1.05 (1.04-1.06) | 1.02 (1.01-1.03) |
| **CALS score** | 1.05 (1.04-1.06) | 1.02 (1.01-1.03) |
| **ACB categories** |  |  |
| 0 (ref) | - | - |
| 1 | 1.04 (1.00-1.07) | 1.02 (0.99-1.06) |
| ≥ 2 | 1.14 (1.11-1.18) | 1.07 (1.04-1.11) |
| **CALS categories** |  |  |
| 0 (ref) | - |  |
| 1 | 1.02 (0.98-1.06) | 1.00 (0.97-1.04) |
| ≥ 2 | 1.13 (1.10-1.17) | 1.05 (1.02-1.09) |

***Model A:** adjusted for age and sex;

****Model B:** adjusted for age, sex, CHF, CAD, COPD, Stroke, Diabetes, Hypertension, atrial fibrillation, cancer, CKD, number of disabled BADL, CPS, Parkinson’s disease, and number of medications at hospital admission**.**

**Supplementary Table 5: logistic regression analysis showing associations between variation of anticholinergic prescriptions during hospital stay and worsening and incidence of dysphagia at hospital discharge**

|  | | **Dysphagia worsening, fully-adjusted model (OR, 95% CI)** | **Dysphagia incidence, fully-adjusted model (OR, 95% CI)** |
| --- | --- | --- | --- |
| **ACB_dis_ - ACB_adm_** | | 0.96 (0.89-1.04) | 1.00 (0.92-1.10) |
|  | **Decreased/Equal** | - | - |
|  | **Increased** | 1.06 (0.82-1.07) | 0.96 (0.68-1.35) |
| **CALS_dis_ - CALS_adm_** | | 0.97 (0.89-1.05) | 0.97 (0.87-1.08) |
|  | **Decreased/Equal** | - | - |
|  | **Increased** | 1.01 (0.78-1.31) | 0.85 (0.60-1.20) |

**Fully-adjusted models:** adjusted for age, sex, CHF, CAD, COPD, Stroke, Diabetes, Hypertension, atrial fibrillation, cancer, CKD, number of disabled BADL, CPS, Parkinson’s disease, and number of medications at hospital admission**.**

**Supplementary Table 6:** logistic regression analysis showing associations between anticholinergic scales and prevalence of dysphagia at hospital admission in patients ≥ 85 years and < 85 years.

|  | **Model B in patients ≥ 85 years** | **Model B in patients < 85 years** |
| --- | --- | --- |
| **ACB score** | 1.02 (1.01-1.03) | 1.03 (1.02-1.04) |
| **CALS score** | 1.02 (1.00-1.03) | 1.03 (1.02-1.04) |
| **ACB categories** |  |  |
| 0 (ref) | - | - |
| 1 | 1.00 (0.95-1.05) | 1.06 (1.01-1.10) |
| ≥ 2 | 1.06 (1.01-1.12) | 1.10 (1.05-1.15) |
| **CALS categories** |  |  |
| 0 (ref) | - | - |
| 1 | 0.98 (0.93-1.03) | 1.04 (1.00-1.09) |
| ≥ 2 | 1.04 (0.99-1.10) | 1.08 (1.04-1.13) |

**Model B:** adjusted for age, sex, CHF, CAD, COPD, Stroke, Diabetes, Hypertension, atrial fibrillation, cancer, CKD, number of disabled BADL, CPS, Parkinson’s disease, and number of medications at hospital admission**.**

**Supplementary Table 7.** Cox Regression analysis showing the association between anticholinergic scales and worsening of dysphagia during hospital stay in patients ≥ 85 years and < 85 years

|  | **Model B in patients ≥ 85 years** | **Model B in patients < 85 years** |
| --- | --- | --- |
| **ACB score** | 1.14 (1.05-1.23) | 1.15 (0.98-1.34) |
| **CALS score** | 1.15 (1.03-1.29) | 1.08 (0.91-1.28) |
| **ACB categories** |  |  |
| 0 (ref) | - | - |
| 1 | 1.38 (0.91-2.09) | 0.70 (0.36-1.27) |
| ≥ 2 | 1.76 (1.18-2.63) | 1.21 (0.67-2.19) |
| **CALS categories** |  |  |
| 0 (ref) | - | - |
| 1 | 1.51 (0.93-2.45) | 0.61 (0.27-1.37) |
| ≥ 2 | 1.73 (1.09-2.05) | 1.32 (0.63.2.76) |

**Model B:** adjusted for age, sex, CHF, CAD, COPD, Stroke, Diabetes, Hypertension, atrial fibrillation, cancer, CKD, number of disabled BADL, CPS, Parkinson’s disease, and number of medications at hospital admission**.**

**Supplementary Table 8.** Cox Regression analysis showing the association between anticholinergic scales and incidence of dysphagia during hospital stay in patients ≥ 85 years and < 85 years

|  | **Model B in patients ≥ 85 years** | **Model B in patients < 85 years** |
| --- | --- | --- |
| **ACB score** | 1.22 (1.09-1.36) | 1.17 (0.98-1.39) |
| **CALS score** | 1.29 (1.10-1.51) | 1.17 (0.95-1.44) |
| **ACB categories** |  |  |
| 0 (ref) | - | - |
| 1 | 1.35 (0.77-2.36) | 1.12 (0.48-2.61) |
| ≥ 2 | 2.26 (1.33-3.85) | 2.01 (0.92-4.38) |
| **CALS categories** |  |  |
| 0 (ref) | - | - |
| 1 | 11.36 (0.72-2.57) | 0.66 (0.22-1.97) |
| ≥ 2 | 2.25 (1.24-4.07) | 1.91 (0.77-4.74) |

**Model B:** adjusted for age, sex, CHF, CAD, COPD, Stroke, Diabetes, Hypertension, atrial fibrillation, cancer, CKD, number of disabled BADL, CPS, Parkinson’s disease, and number of medications at hospital admission**.**
